# Proffering Connections: Psychologising Experience in Psychotherapy and Everyday Life

**DOI:** 10.3389/fpsyg.2020.583073

**Published:** 2021-01-14

**Authors:** Stuart Ekberg

**Affiliations:** ^1^School of Psychology and Counselling, Queensland University of Technology, Brisbane, QLD, Australia; ^2^Centre for Healthcare Transformation, Queensland University of Technology, Brisbane, QLD, Australia

**Keywords:** conversation analysis, everyday conversation, psychotherapy, non-specific benefit, reference, connections

## Abstract

Conversation analytic research has advanced understanding of the psychotherapeutic process by understanding how psychotherapy is organised over time in and through interaction between clients and therapists. This study progresses knowledge in this area by examining how psychological accounts of experience are progressively developed across a range of helping relationships. Data include: (1) approximately 30 h of psychotherapy sessions involving trainee therapists; (2) approximately 15 h of psychotherapy demonstration sessions involving expert therapists; and (3) approximately 30 h of everyday conversations involving close friends or family members. This article reports an analysis of techniques that are used to bring together two experiences that were discussed separately, to proffer a candidate connection between them. This proffering of candidate connections was recurrently used in psychotherapy. If confirmed by a client, a proffered connection could be used to develop a psychological account of a client’s experiences, which could then warrant some psychological intervention. In contrast, the proffering of connections was observed in only one of the everyday conversations included in the current study, where it was used to develop psychological accounts of experience. This shows that although proffering candidate connections is an everyday interactional practice, it appears to be used with greater frequency in psychotherapy, to advance its specific institutional aims.

## Introduction

“…it might be conceivably argued that psychoanalysis…succeeds, when it does…because the analyst, in the practice of his method, quite unwittingly allows the patient to recondition certain inadequate social patterns in terms of the present situation” (Rosenzweig, 1936: 412)

Psychotherapy, at least in its traditional, mainstream, and predominant senses, is a fundamentally interpersonal and interactive endeavour. In contrast to biomedical treatments, such as pharmacology, psychotherapeutic interventions typically involve social encounters where individuals or groups experience sustained reflective engagement about their mental disorders, problems, or complaints. The ultimate goal of psychotherapy is to transform, in some way, an individual’s or group’s experience to help alleviate a disorder, problem, or complaint (Wampold and Imel, 2015). Even when psychotherapeutic interventions are not interpersonal, such in bibliotherapy and computerised treatments (Marrs, 1995; Grist and Cavanagh, 2013; Eells et al., 2015), there are nonetheless interactive encounters between a person and a therapeutic medium that are intended to sustain reflective engagement. Interaction thus appears to be a central part – or even *the* central part – of psychotherapy. Nevertheless, crucial aspects of interaction have been overlooked in attempts to understand the psychotherapeutic process. This study aims to further understanding of psychotherapy through fine-grained analysis of its moment-by-moment production in and through social interaction.

Although some definitions of psychotherapy acknowledge that interaction between a therapist and client provides a medium for therapy (e.g., Jørgensen, 2019: 26), psychotherapy research does not typically emphasise comprehensive exploration of how therapy is produced in and through these social encounters. For example, recent reviews conducted by the American Psychological Association’s Task Force on Evidence-based Relationships and Responsiveness (Norcross and Lambert, 2019) highlight a range of relational practices that are accomplished in and through social interaction. Nevertheless, these reviews do not specifically acknowledge social interaction as a bedrock of psychotherapy. Social interaction provides the infrastructure necessary for the accomplishment of social institutions such as psychotherapy, as well as constituting the primordial site of human sociality more generally (see Schegloff, 2006). It is therefore necessary to understand the details of social interaction to understand the psychotherapeutic process.

One field of research specialising in the study of social interaction, conversation analysis, has been increasingly applied to the study of psychotherapy (Madill et al., 2001; Peräkylä et al., 2008; Buchholz and Kächele, 2013; Peräkylä, 2013, 2019; Madill, 2015; Buchholz, 2017). Focusing, in detail, on the moment-by-moment progress of social interaction, conversation analysis provides means for understanding how “…psychotherapeutic processes are embedded in the concrete details of social interaction” (Peräkylä, 2019: 278). The current study contributes to this analytic enterprise, focusing on ways clients and therapists progressively establish psychological accounts of experience that align with the goals of psychotherapy.

To date, most conversation analytic research investigating psychotherapeutic encounters has focused on ways participants organise these encounters into sequences of action (Peräkylä, 2019). Both in psychotherapy and social interaction more generally, organising actions into sequences enables participants to understand a current action in relation to the actions that precede it, as well as in relation to what a current action may make relevant as potential next actions (Schegloff, 2007). For instance, the following fragment from a psychotherapy session is organised into two sequences.

In this fragment, both sequences are initiated by the therapist, and each is designed differently to implement particular actions. The therapist’s first turn (lines 1–2) is designed as a question about the client’s experience of ‘visions’, namely whether they occur less frequently when he is with his parents. The client confirms this by explaining why he does not experience as many visions in this context (lines 4–5). In contrast to this first sequence, which is initiated by the therapist’s question, the second sequence (lines 6–7) is initiated by an ‘upshot formulation’ (Antaki et al., 2005). This formulation exposes something implied, although not stated, in the client’s prior turn. This enables the therapist to highlight an aspect of the client’s problem: namely, that he is more likely to experience visions when alone and inactive. Through two short sequences – a question-response sequence followed by a formulation-confirmation sequence – the therapist and client progress toward a particularised and psychological account of the client’s experience. Instances like this demonstrate how, at a fundamental level, understanding the organisation of social interaction is essential for understanding the psychotherapeutic process, which “takes place *through these sequences*” (Peräkylä, 2019: 265, emphasis added).

Beyond revealing the fundamental importance of sequence organisation for understanding how psychotherapy progresses, recent conversation analytic research has incorporated a broader perspective. One avenue of inquiry involves extending analysis beyond relatively short sequences of action, such as in Fragment 1, to focusing on the organisation of psychotherapy over longer periods of time (Voutilainen et al., 2011, 2018; Bercelli et al., 2013; Buchholz and Kächele, 2017). This level of organisation appears to involve alternating periods of enquiry (Bercelli et al., 2008, 2013), where therapists and clients work to recognise relevant aspects of the client’s circumstances (Voutilainen et al., 2010b), and elaboration (Bercelli et al., 2008, 2013), where the parties are predominantly focused on interpreting those circumstances (Voutilainen et al., 2010b). Through this process, the understanding of some matter that is a focus within therapy, such as feelings of blame (Voutilainen et al., 2011), can be progressively understood and transformed over time. This research shows how, over time, participants collaboratively produce particular versions of a client’s experience (Peräkylä, 2019).


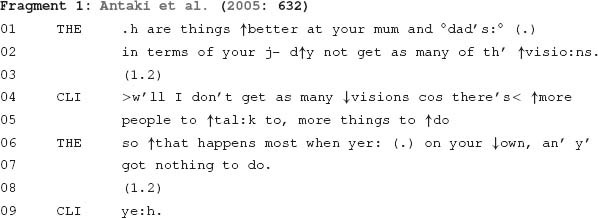


A second way in which conversation analytic research has understood the organisation of psychotherapy over time is through an examination of the use of referential practices (Clark and Rendle-Short, 2016; Buchholz and Kächele, 2017; Voutilainen et al., 2018; Peräkylä, 2019). Through reference to a variety of entities from diverse ‘ontological domains’ (Enfield, 2013) – such as people, places, objects, time, and conduct – participants can focus on particular referents for particular purposes. For example, Voutilainen et al. (2018) show how repeated used of the reference ‘dominant’ connects one discussion about a client’s sister-in-law to a current discussion about the client herself. Foundational interactional processes, such as reference, can thus become incorporated into accomplishing the business of psychotherapy.

By considering the organisation of psychotherapy over time, conversation analytic research is progressively understanding how the psychotherapeutic process helps clients overcome the difficulties that brough them to therapy (Peräkylä, 2019). Sustained research is needed in this area to continue specifying the precise ways diverse psychotherapeutic approaches share a capacity to alleviate clients’ difficulties (Rosenzweig, 1936; Rosenthal and Frank, 1956; Stiles et al., 1986; Wampold and Imel, 2015). Sustained research will also inform understanding how psychotherapy differs – if it differs at all – from other types of ‘helping relationships’ (Rogers, 1958), such as those that one might have with a close friend or family member (Siegfried, 1995; Kozart, 1996; Mondada, 1998; Pain, 2009; Pawelczyk, 2011; Jørgensen, 2019). The current study is designed to address these matters by exploring how psychological accounts of experience are progressively developed in conversations that occur across different types of helping relationships.

## Data and Methods

This comparative conversation analytic study (Drew and Heritage, 1992; Sidnell, 2009) involved video recording both psychotherapeutic and everyday – or mundane – interactions that occurred in Australia. Ethical clearance was provided by the Queensland University of Technology (QUT) Human Research Ethics Committee for both the psychotherapeutic (Approval reference: 1600001155) and mundane data (Approval reference: 1600001058). Each party to a recorded interaction was independently informed about the study and asked whether they were willing to consent to participate. Data were only collected if each party freely and independently consented to participate.

Mundane data were collected in a variety of settings, including private homes and public spaces (e.g., parks). Each interaction involved a small group of friends or family members. Approximately 30 h of interaction were video recorded across 20 dyads and 2 triads. Psychotherapeutic interactions were video recorded within a single clinic that is predominantly staffed by trainee psychotherapists. This clinic specialised in a range of different psychotherapeutic approaches. Table 1 reports the therapeutic approach that therapists explained to clients as the predominant approach they were adopting. Nevertheless, correspondence between the researcher and trainee therapists indicated that many moved beyond a single psychotherapeutic perspective to adopt an ‘integrative approach’ (Norcross and Goldfried, 2019). Approximately 30 h of recorded psychotherapeutic interaction was examined across four therapist-client dyads.

**TABLE 1:** Participant details for psychotherapy data.

Dyad	Predominant Approach
Male client, female therapist	Interpersonal Therapy
Female client, female therapist	Psychodynamic Psychotherapy
Male client, female therapist	Cognitive Behavioural Therapy
Female client, female therapist	Schema Therapy

Psychotherapeutic data were collected during the first year of clinical practice for trainee psychotherapists, when they were still learning fundamental aspects of different therapeutic approaches. Focusing on trainees rather than more experienced therapists foregrounds the therapeutic context rather than the skill of individual therapists. By comparing therapeutic interactions involving trainee therapists with mundane interactions involving friends or family members, the study aims to examine how interactional practices are designed, either in similar or different ways, to suit the institutional contexts of psychotherapy and the everyday contexts of mundane interaction.

Although the focus of the current study relates to the therapeutic context rather than the skill of individual therapists, a secondary aim of the study was to examine whether the interactional practices that comprise the focus of the study are restricted to trainee therapists or might also comprise the practice of more experienced therapists. To fulfil this aim, additional data were obtained from demonstration sessions of therapy conducted by expert psychotherapists. Most of the database is comprised of recordings made in the United States of America. Approximately 15 h of these demonstration sessions were sourced from the Counseling and Therapy in Video database published by Alexander Street Press, a source of data previously used for conversation analytic research (e.g., Kondratyuk and Peräkylä, 2011; Muntigl and Horvath, 2014).

The study used typical methods for conversation analytic research (Sidnell, 2013). Analysis commenced with a phase of ‘unmotivated examination’ of the psychotherapy data. Using this unmotivated approach, rather than guiding analysis by psychotherapeutic theory, provided opportunities to notice phenomena that might not be foreground or otherwise anticipated by such theories (Sacks, 1984; Peräkylä and Vehviläinen, 2003; Madill, 2015; Voutilainen and Peräkylä, 2016). Specialised transcription conventions for spoken (Hepburn and Bolden, 2013) and embodied conduct (Mondada, 2018) were employed to facilitate detailed analysis (please refer to the Appendix for a transcription key). The names of all participants, and any third parties mentioned in the data, were replaced with pseudonyms.

In the present study, the unmotivated examination phase of analysis resulted in identification of a recurrent practice where one party cited some prior conduct. These citations could relate to the conduct of another (e.g., “You said…”), oneself (e.g., “I mentioned…”), some group (e.g., “We discussed…”), or objects such as documents (e.g., “It says…”). These instances were gathered into a collection (Sidnell, 2013), which was progressively expanded through analysis of the mundane data in addition to the psychotherapeutic data. A separate report describes the generic properties of citing sources (Ekberg, Unpublished; see also Goffman, 1974, 1979; Pomerantz, 1984). The study reported here focuses specifically on the similarities and differences between the use of this practice across psychotherapeutic and mundane interactions.

## Analysis

### Proffering Connections in Psychotherapy Sessions

In their attempts to progressively understand mental disorders, problems, or complaints, psychotherapists and clients dedicate considerable time, over many psychotherapy sessions, discussing the client’s circumstances. Over time, an increasingly shared understanding of these circumstances enables either of these parties – although usually the therapist – to proffer candidate connections between experiences that have been discussed at different times within a session, or even across different sessions. The following analysis will consider the use of ‘locational tying techniques’ that are used to invoke some past utterance as relevant for a present discussion (Sacks, 1995). These techniques facilitate bringing together two or more things that have been previously discussed, but not in relation to one another. Once these matters are co-located, a current speaker – usually the therapist – can pursue a course of action that involves a connection between these matters. This connection is typically a candidate one, and so the recipient – usually the client – may confirm or reject it. If confirmed, these connections can facilitate the progressive establishment of psychological accounts of clients and their circumstances.

The following fragment is an instance where a locational tying technique is used to proffer a candidate connection between two matters that have not been discussed in relation to one another. It comes from a fourth session of psychotherapy involving a male client and female therapist (Dyad 1 in Table 1). It begins with the client making a claim about his tendency to exhibit emotional detachment. During this discussion, the therapist proffers a connection between what the client is currently discussing and something he mentioned two sessions previously. This previous mentioning is introduced through use of a locational tying technique.


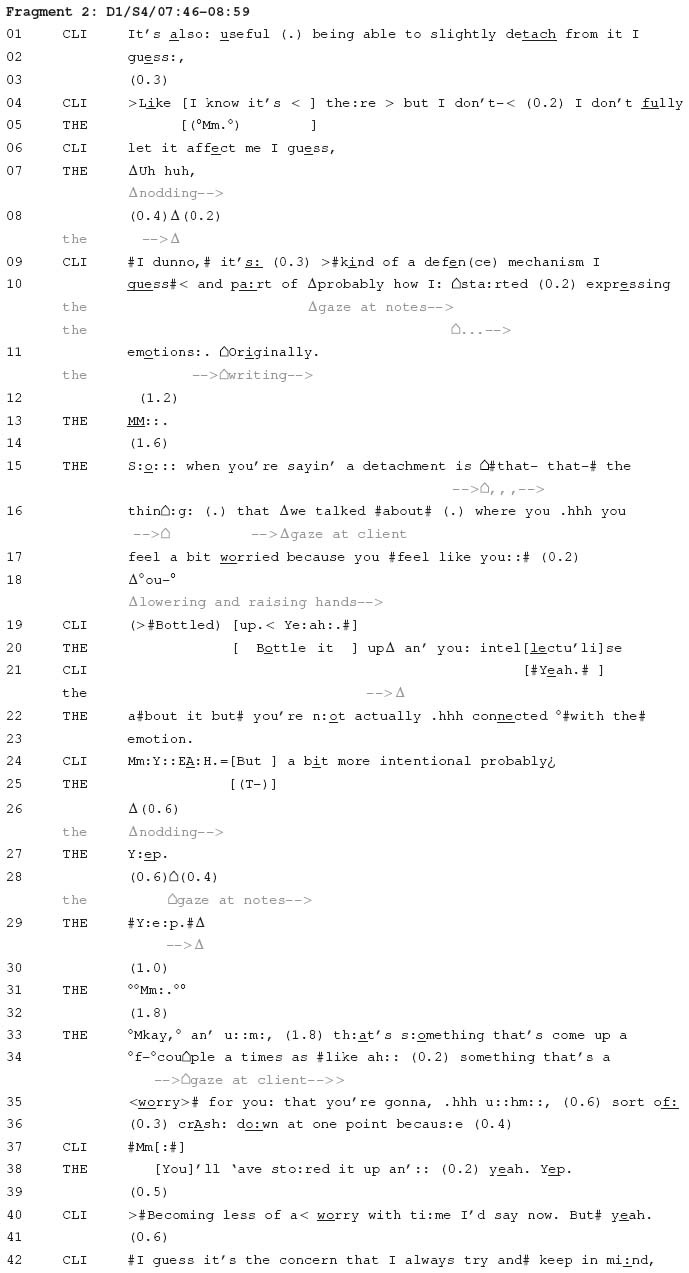


In this fragment, the therapist seeks to clarify the meaning of the client’s term ‘detach’ (line 1), which she attributes to something the client has recently said: “when *you’re saying* a detachment” (line 15, emphasis added). To make this clarification, she refers to something about which the client is ‘worried’ (line 17). The design of the therapist’s term includes citing the source of this claim (Ekberg, Unpublished; Goffman, 1974, 1979; Pomerantz, 1984). She does this by prefacing her reference to ‘worry’ with an description of it as “the thing that we talked about” (lines 15–16). This citing of a source functions as a locational tying technique, invoking something that occurred elsewhere as relevant for the present (Sacks, 1995). The technique indicates that what is being discussed now – detachment – is going to be connected to something this dyad have discussed before.

Before the therapist specifies the connection that she is making between the present and a past discussion (lines 15–18), the client completes the therapist’s turn to specify “the thing that we talked about” (line 16) as “bottled up” (line 19). The client’s expression may be a vocalisation of the therapist’s hand gesture (lines 18–20), as “bottled up” has not actually been used in previous sessions. The client did, however, mention in his second session of therapy that he intellectualises his emotions, and the therapist refers to this in her response (lines 20–23). By this point in their conversation, the therapist has used a locational tying technique to bring together two matters that were raised separately. In doing so, she is able to ask a question about a candidate connection between them: “So when you’re saying a detachment is that the thing that we talked about where you feel a bit worried because you feel like you…bottle it up and you intellectualise about it but you’re not actually connected with the emotion” (lines 15–23). The therapist orients to the client’s epistemic primacy in this matter (Heritage, 2012), making this connection within a question that seeks the client’s confirmation or rejection as a relevant next action. What has been proffered here therefore remains a candidate connection until it is confirmed by the client.

Following the client’s qualified endorsement of this connection (line 24), the therapist then claims that the client’s tendency to detach or intellectualise has come up before in therapy, and is connected to the client’s concerns about this contributing to periodic emotional breakdowns (lines 33–38). In contrast to the first connection, which was proffered to the client through a question, this second connection is instead asserted by the therapist. The client nonetheless treats this as a matter over which he has epistemic primacy, providing a qualified endorsement of the connection the therapist has made (line 40). It is in this sense that these candidate connections can be understood as proffered. They are presented by one party to another, for the recipient to ultimately confirm or reject.

In this fragment, the therapist proffers a candidate connection between one matter – the client’s detachment – and another matter discussed earlier in therapy – the client’s intellectualising. The therapist then goes on to proffer a broader candidate connection between these experiences and the client’s risk of emotional breakdown. This proffering of connections appears to be a common undertaking in therapy, occurring in many therapy sessions. If this candidate connection comes to be accepted by the client, this may then inform the dyad’s psychological account of the client and their circumstances. The next fragment illustrates this process.

The following fragment is another instance where a candidate connection is proffered across matters that have been discussed within therapy. It comes from a tenth session of psychotherapy involving a female client and female therapist (Dyad 4 in Table 1). The fragment comes midway through discussion of the origin of the client’s belief that her relationships will eventually fail, because people will abandon her once they come to know her personality and health challenges (the latter is referred to in the fragment as ‘fibro’, for fibromyalgia). The therapist is sitting next to the part of a whiteboard that she has written on. Her writing documents key points about a discussion that has continued up until the beginning of this fragment. This part of the whiteboard is approximately forty-five degrees counter clockwise from the therapist’s overwhelming physical orientation towards the client. Due to her physical position, the therapist rotates her head to shift her gaze between the client and the whiteboard throughout the fragment. She also refers, on multiple occasions, to two beliefs that the client holds, which are written next to one another on the whiteboard: (1) “If people know me they will leave”; (2) “If I am myself they will leave.” Through both her verbal and physical conduct, the therapist proffers a candidate connection in which these beliefs underpins the client’s behaviour with different types of people. The fragment begins following the therapist’s question about whether the client’s experiences of abandonment go beyond recent experiences to include her time at school.


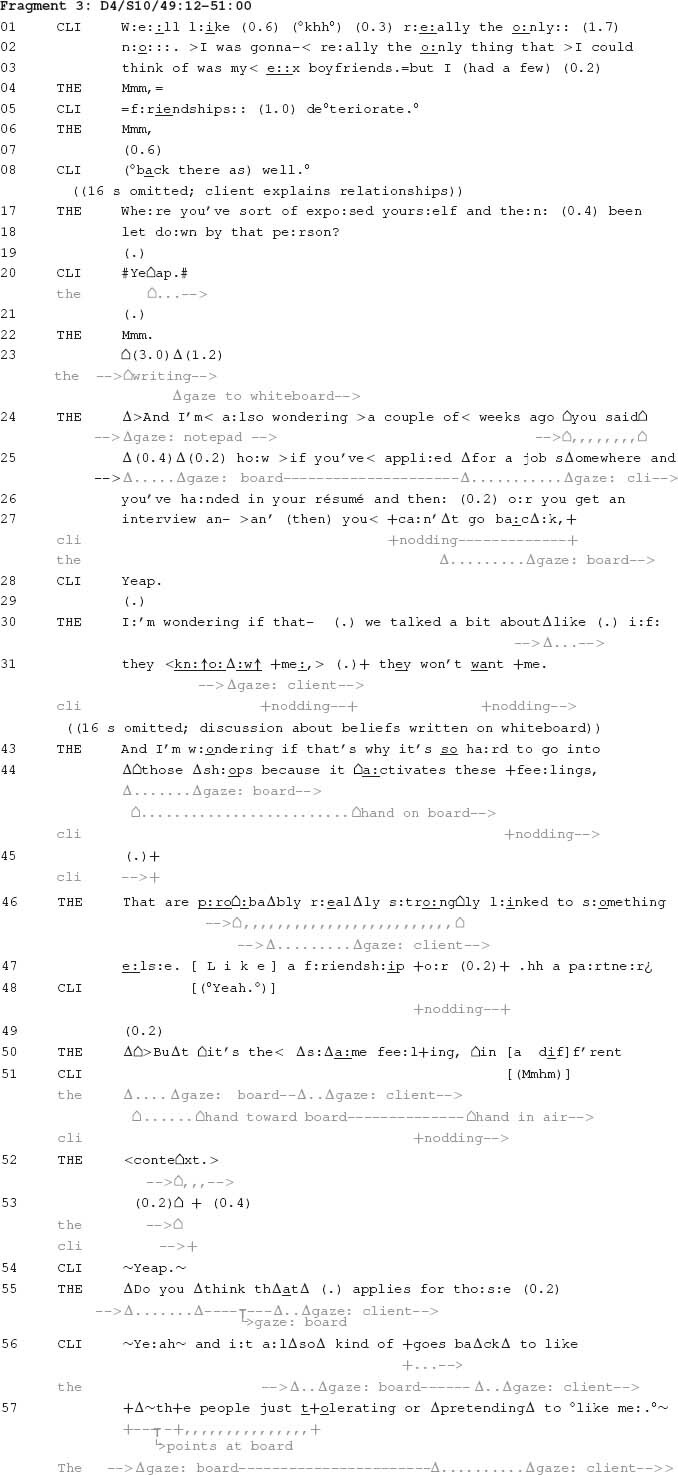


The fragment begins with discussion about the history of the client being abandoned by others, which has focused on close friendships and romantic relationships. Then, at line 24, the therapist specifies the source of her upcoming claim (“a couple of weeks ago you said”). Using this locational tying technique invokes something discussed in a previous therapy session, about how the client cannot return to shops where she has unsuccessfully applied for work. Following this, the therapist uses another locational tying technique to return to aspects of their more recent discussion in this session: “we talked a bit about like if they know me, they won’t want me” (lines 30–31). By bringing together things that were previously mentioned separately, the therapist can ask a question about whether there is a connection between them. This is subsequently expanded further by the therapist, who asks a question that connects the client’s beliefs about shops with relatively intimate types of interpersonal relationships such as with “a friendship or partner” (lines 43–47). A connection is thus proffered where beliefs about abandonment, which have been developed in the context of close interpersonal relationships, are used to explain why the client is not able to return to shops where she has unsuccessfully applied for work. In contrast to Fragment 2, which contained more qualified confirmation, the candidate connection proffered in this fragment receives much stronger confirmation from the client (e.g., lines 56–57). This confirmation facilitates psychological activities that are pursued subsequently.

Through proffering a candidate connection based on beliefs about abandonment, the therapist proposes a psychological account of the client’s experience. This is accepted by the client, who subsequently expands this connection to include other matters the dyad have discussed (lines 56–57). In the following session (data not shown), the therapist resumes this discussion, referring to it as a discussion of an ‘abandonment schema.’ She uses this to initiate informing the client about schema therapy, the predominant approach used in her sessions with the client (see Table 1). The therapist continues to explain how she hopes to use this to change how the client relates to schemata such as the abandonment schema. The proffering of a connection thus provides a basis for advancing a psychological account of the client’s challenges, and then a potential psychological solution.

The instances considered to this point relate to adverse experiences. The focal practice can also be used to highlight a client’s strengths. The next fragment is one such instance. It comes from an eighth session of therapy involving a female client and female therapist (Dyad 2 in Table 1). The client is attending therapy due to depression and anxiety. The fragment begins partway through a discussion about a period of intense anxiety the client experienced as a child. Across this fragment, the therapist proffers a connection between what the client reports happening during her childhood and what she is now experiencing as an adult.


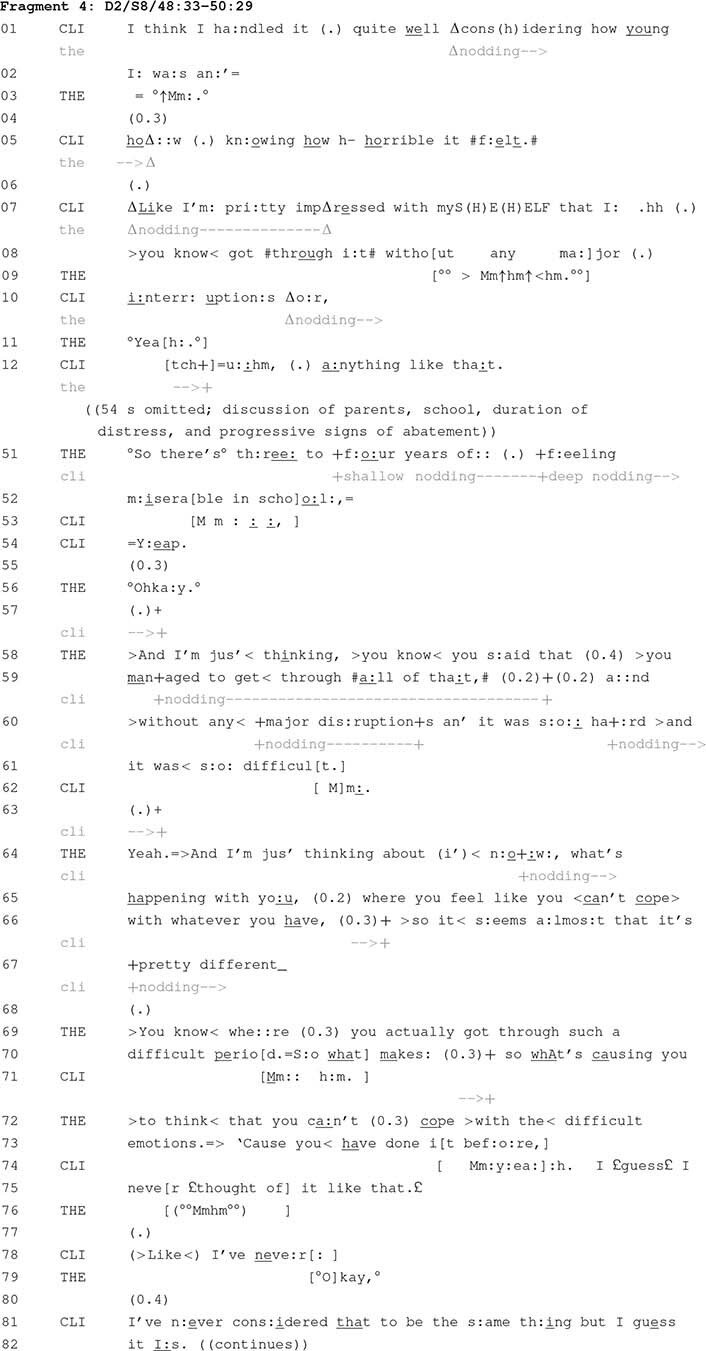


Toward the beginning of this fragment, the client mentions her resilience during a period in her childhood when she experienced intense anxiety: “I think I handled it quite well” (line 1). In the moments that follow (data not shown), the discussion moves away from this specific focus on the client’s ability to manage her anxiety. The therapist, however, subsequently brings the discussion back to this matter. She does this by producing an upshot formulation that focuses on the duration of the client’s period of anxiety (lines 51–52). Following the client’s confirmation of this (lines 53–54), the therapist then cites something the client mentioned even earlier: “you said that you managed to get through all of that, and without any major disruptions” (lines 58–60). This locational tying technique makes relevant the client’s resilience during a period of her childhood when she experienced intense anxiety, so it can be considered in relation to the current discussion of the client’s contemporary challenges.

Having returned the focus of discussion to the client’s resilience during a period of past anxiety, the therapist connects this with a matter that has been a focus of both this and prior therapy sessions: “And I’m just thinking about it now, with what’s happening with you, where you feel like you can’t cope with whatever you have” (lines 64–66). This makes a connection between two periods in the client’s life during when she has been confronted with intense anxiety. This connection enables the therapist to highlight a difference in the client’s understandings of those periods. As with the second connection in Fragment 2 (lines 33–40), this connection is asserted by the therapist (“what’s causing you to think that you can’t cope with the difficult emotions because you have done it before”, lines 70–73). As was also the case in Fragment 2, the client nonetheless treats this assertion as something to be confirmed. It is in this sense that the connection can be understood as a candidate one, and moreover one that has been proffered for confirmation or rejection.

By proffering a candidate connection between the client’s childhood and her present, both periods during which she has reported experiencing extreme mental distress, the therapist warrants asking the client why she was able to cope in the past but does not appear to be able to do so in the present (lines 70–73). Developing an understanding of this has the potential to enable the client and therapist to conceptualise both the challenges that confront the client, as well as the resilience that she might employ to address these challenges. This exploration is accomplished by connecting different experiences the client has reported at particular points of the therapeutic process. This proffers a connection that may come to comprise a psychological account of the client, her experiences, and her capacity for resilience.

The above fragments highlight ways trainee psychotherapists proffer connections between experiences that the therapist and client have discussed at particular points of the therapeutic process. This enables therapists to bring together experiences that were not previously connected to consider, with the client, whether there is indeed some connection between those experiences. This practice does not appear to be restricted to trainee psychotherapists. As the following fragment demonstrates, highly experienced therapists also employ this practice to proffer candidate connections between clients’ experiences. This fragment starts approximately seven minutes into a therapy session involving a male client and male therapist. Before coming to therapy, the therapist asked the client to complete a 15-page multimodal life history inventory (Lazarus and Lazarus, 1991). The therapist has read the client’s completed inventory prior to the beginning of this session and refers to this during a focal moment in the fragment that follows.


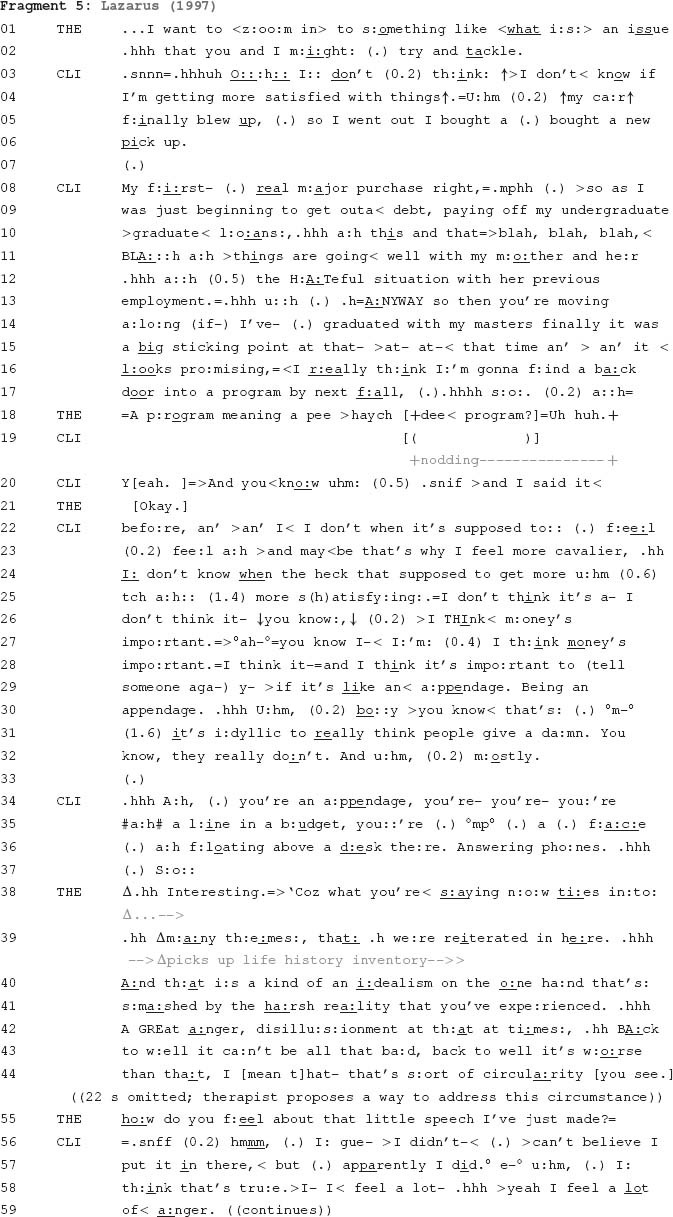


In this fragment, the client moves from describing circumstances that provide a potential for satisfaction (lines 3–17) to circumstances that are depicted as intractable obstacles to satisfaction (lines 20–37). From line 38 the therapist connects what the client has just been verbally describing and what the client had written in his life history inventory. He does so by picking up the inventory and citing it as the source of his claim (“themes that were reiterated in here”, line 39). By using this technique, the therapist brings together two things that the client has expressed separately: in his current discussion with the therapist and when completing the life history inventory. Having brought these two matters together, the therapist produces an explanation that proffers a connection between them: “a kind of an idealism on the one hand that’s smashed by the harsh reality that you’ve experienced” (lines 40–41).

The therapist culminates his extended turn by underscoring its candidate status, asking the client about the appropriateness of the explanation he has proffered (line 55). The client’s response confirms that the therapist’s explanation connects these matters (lines 57–59). Thus, very early in this dyad’s relationship, a connection between two matters that were raised separately come to be proffered by the therapist and accepted by the client. Here in Fragment 5, a psychotherapy session involving an expert psychotherapist, and above, in fragments from sessions involving trainee psychotherapists, such proffering of candidate connections facilitates the progressive establishment of psychological accounts of clients and their circumstances. This type of activity is thought to be a fundamental aspect of psychotherapy (Jørgensen, 2019). As the next section shows, however, psychotherapy is not the only context where this occurs.

### Proffering Connections in Mundane Interaction

In contrast to psychotherapeutic interactions, the use of locational tying techniques to proffer candidate connections between experiences was much less common in the mundane interactions that were recorded for the current study. The following fragment, however, comes from one conversation that is an exception. Throughout this conversation there are numerous attempts by Peter to proffer candidate connections in relation to Dean’s interpersonal experiences. Each of these instances relates to a focus these two men have on discussing trauma that Dean has apparently experienced at some point in his past. The fragment begins with Peter summarising a section of a book that he has previously shown to Dean when they were travelling together on a bus. Apparently they were not able to discuss this section of the book openly in a public place, so Peter seeks to resume their discussion of it now.


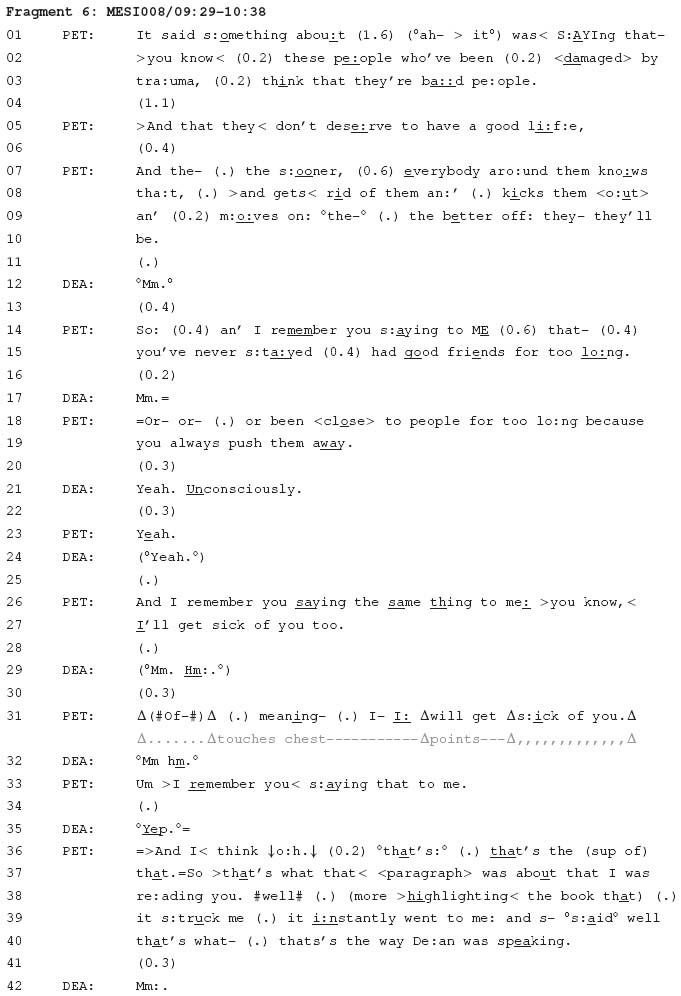


In this fragment, Peter and Dean are discussing a psychological topic: trauma. Peter refers to claims about trauma that are made in a book he has been reading (see, for example, line 1). He then connects this with things that he claims Dean has said in the past about his interpersonal experiences (e.g., lines 14–15). Consistent with previous observations in psychotherapy (Bercelli et al., 2013; Weiste and Peräkylä, 2013), citing something said in the past can be a locational tying technique to place prior conduct in a new context where it can take on a different meaning. Here, by citing Dean’s prior conduct, Peter can proffer a candidate connection between that conduct and what the book claims about people who have experienced trauma.

Citing Dean’s prior conduct provides means for Peter to proffer a connection between Dean’s interpersonal experiences, and thereby facilitate a psychological account of those experiences: namely, that Dean behaves in a similar way to the manner in which this book claims people with trauma tend to behave. A similar observation has been by Arminen (1998) in a different therapeutic context, Alcoholics Anonymous, where connections are made between a member’s experience and an experience described on a television program. Across that context and the context of the current study, this practice is used in a comparable way: to proffer a candidate connection between two or more experiences. This proffering of candidate connections was recurrently observed in therapy, but was far less common in the mundane interactions that were recorded for the current study.

## Discussion

Existing attempts to explain how psychotherapy works tend to focus exclusively on therapeutic interaction, rather than comparing these encounters with other types of social interaction. This may be one reason for the longstanding difficulty in determining how diverse approaches to therapy share a common capacity to alleviate mental distress (Rosenzweig, 1936; Rosenthal and Frank, 1956; Stiles et al., 1986; Wampold and Imel, 2015). Although some psychotherapy researchers consider psychotherapy as a social or cultural practice (e.g., Wampold and Imel, 2015; Jørgensen, 2019), ongoing research is needed to understand what distinguishes therapy from social or cultural practices that occur in other contexts (Siegfried, 1995; Kozart, 1996; Mondada, 1998; Pawelczyk, 2011; Jørgensen, 2019). Comparative conversation analytic research affords opportunities to explore this matter (Drew and Heritage, 1992).

It is possible that the bulk of therapeutic encounters are comprised of mundane interactional practices (Mondada, 1998), which are used in ways that suit the particular roles and activities that comprise this institutional activity (Lakoff, 1982; Drew and Heritage, 1992; Pain, 2009; Pawelczyk, 2011). In recognition of this possibility, the current study involved directly comparing psychotherapeutic and mundane interaction. The current study identifies a practice that is relatively pervasive in psychotherapy but appears to be much less common in mundane interaction. This practice involves use of locational tying techniques to bring together two or more experiences that were independently discussed, enabling a candidate connection to be proffered between them. In instances where a candidate connection is confirmed, this can facilitate the production of a psychological account of such experiences. In psychotherapy, as observed above in the analysis of Fragment 3, the production of a psychological account of a client’s difficulties can then facilitate the pursuit of a psychological solution.

The findings of the current study are congruent with similar conversation analytic studies of psychotherapy (Wowk, 1989; Parker, 2003; Vehviläinen, 2003; Peräkylä, 2004). Although the foci of these studies are somewhat different to the present study, each considers ways therapists connect different experiences reported by clients, often across multiple therapy sessions. The current study supports and extends this analysis. In particular, by comparing ways candidate connections are proffered across psychotherapeutic and mundane interaction, the current study highlights how this practice can be used to psychologise experience. This is consistent with findings by Arminen (1998), who considers how connections are made between the experiences of different people participating in Alcoholics Anonymous (see also Halonen, 2008). Taken together, the findings of these previous studies and the current study suggest that proferring connections can be used to psychologise people individually or collectively. Moreover, this practice can occur across a range of ‘helping relationships’ (Rogers, 1958), although it seems to be used more frequently in therapeutic settings, where this practice contributes to core activities for psychotherapy.

More generally, the results of the current study are consistent with the perspective that parties to psychotherapeutic encounters are recurrently engaged in activities that share a generic focus on systematically identifying possibly relevant aspects of experience (Bercelli et al., 2013). Along with similar studies (Arminen, 1998; Parker, 2003; Vehviläinen, 2003; Peräkylä, 2004; Halonen, 2008; Pain, 2009), the current study finds that exploration of possibly relevant aspects of experience routinely involves attempts to connect experiences. If such connections can be made, these can form the basis of a psychological account of experience, which can then underpin subsequent therapeutic work.

Identifying candidate connections is theorised to be a central activity for psychotherapy (Tomm et al., 2014; Jørgensen, 2019). The current study provides evidence of this, and highlights how this can distinguish therapy from other types of social encounters. This finding is congruent with transtheoretical views of psychotherapy, such as understanding a “…patient’s reports as meaningful stories to be interpreted and modified in collaboration with the therapist” (Frank and Frank, 1991: 73). As reflected in the quote at the beginning of the article, this finding is also consistent with longstanding recognition that a core part of psychotherapy is the identification and reconditioning of ‘patterns’ (Rosenzweig, 1936). In the ongoing effort to understand such common factors that seem to underpin the success of a diverse range of therapeutic processes (Rosenzweig, 1936; Rosenthal and Frank, 1956; Stiles et al., 1986; Wampold and Imel, 2015), the type of comparative work undertaken in the current study is important to identify social practices that help answer this enduring question.

According to prior conversation analytic research (Voutilainen et al., 2010b; Bercelli et al., 2013), psychotherapeutic encounters appear to be organised according to general types of interactional projects (Schegloff, 2007). This level of organisation appears to involve alternating periods of enquiry (Bercelli et al., 2008, 2013), where therapists and clients work to recognise relevant aspects of the client’s circumstances (Voutilainen et al., 2010b), and elaboration (Bercelli et al., 2008, 2013), where the parties are predominantly focused on interpreting those circumstances (Voutilainen et al., 2010b). The current study has focused on a practice that provides means to integrate enquiry and elaboration. Doing so has highlighted the potentially protracted nature of this process, which routinely involves proffering candidate connections between experiences that were separately mentioned across several therapy sessions. This is consistent with the observation by Bercelli et al. (2013) that psychotherapy is characterised by alternation between different interactional projects. The current study identifies one practice through which this alternation is accomplished.

The alternation between different interactional projects in psychotherapy distinguishes this type of interaction from other types of institutional interaction. For example, primary care consultations for acute medical conditions are characterised by a typically liner progression through a series of interactional projects, or ‘phases’ (Robinson, 2003). In contrast, psychotherapy appears to be characterised by more non-linear progression, such as by returning to past matters that may be relevant to a current activity.

At a more general level, alternation between different interactional projects also distinguishes psychotherapeutic from mundane interaction. In mundane settings, talk about troubles tends to be oriented to by participants as an episodic activity, and one which is routinely closed so the parties can return to ‘business as usual’ in which troubles are not the focus of their interaction (Jefferson, 1984, 1988; Holt, 1993; Maynard, 1997, 2003). In contrast, talking about troubles *is* the usual business of therapy (Davis, 1986; Ferrara, 1994; Ruusuvuori and Voutilainen, 2016; Jørgensen, 2019), even though troubles may not be discussed in the same way in psychotherapy as it is in mundane interaction (see Voutilainen et al., 2010a). With the exception of mundane interactions such as the one considered above, a crucial difference between mundane and psychotherapeutic interactions is that the latter involves sustained focus on troubles over a multitude of encounters (Ferrara, 1994; Voutilainen et al., 2018). This sustained focus on an individual and their experiences is a key point of difference between the diverse activities that are likely in mundane social interactions. This may account, at least in part, for the recurrent use of the focal practice in psychotherapy in contrast to its relatively more scare use in mundane interaction. The current study shows how proffering candidate connections is one way therapists and clients can sustain focus on the client’s troubles across the psychotherapeutic process.

There are several limitations to the current study that should be considered when interpreting findings and planning future research. First, the study used data collected from a small number of therapists working from a range of psychotherapeutic approaches. Although this allowed identification of an interactional practice that occurs across this diversity, there was no scope to examine different therapists who claimed to use the same therapeutic approach. Second, the current study was limited in focus to dyadic psychotherapy involving individual clients and therapists. Further research will be necessary to determine whether these findings are transferrable to other types of therapeutic encounters, such as with groups of people or conducted via alternative media, such as computerised treatments. Third, it is possible the practice of proffering candidate connections was originally developed for psychotherapy and subsequently appropriated in everyday contexts for purposes such as psychologising. That is, rather than being a mundane interactional practice that is used to accomplish the business of psychotherapy, it is possible that this practice originally developed in psychotherapy and has been subsequently adopted for use in mundane settings (see Pawelczyk, 2011). Notwithstanding these limitations, the current study illustrates the promise that comparative research holds for understanding what unites diverse approaches to psychotherapy and how this might be distinct from other types of supportive encounters.

If psychotherapy is indeed “an unusual social relationship” (Wampold and Imel, 2015: 56), the current study, along with existing conversation analytic research, helps to understand the precise ways therapy differs from what typically occurs in other types of social encounters. The findings of this study highlight ways that everyday interactional practices appear to be adapted to suit the local context of psychotherapeutic encounters. In psychotherapy, the repeated use of practices, such as using locational tying techniques to proffer candidate connections between experiences, provides means for therapists and clients to progressively develop a psychological understanding of the client and their circumstances. This understanding may ultimately contribute to meeting the needs that motivate clients to participate in therapy.

## Data Availability Statement

The datasets presented in this article are not readily available because data sharing is not permitted under the ethical clearances for this study. Requests to access the datasets should be directed to SE (stuart.ekberg@qut.edu.au).

## Ethics Statement

The studies involving human participants were reviewed and approved by Queensland University of Technology (QUT) Human Research Ethics Committee. The patients/participants provided their written informed consent to participate in this study. Written informed consent was obtained from the individual(s) for the publication of any potentially identifiable images or data included in this article.

## Author Contributions

The author confirms being the sole contributor of this work and has approved it for publication.

## Conflict of Interest

The author declares that the research was conducted in the absence of any commercial or financial relationships that could be construed as a potential conflict of interest.
